# Finding the right fit: How mentor-mentee fit impacts outcomes for academic mentees

**DOI:** 10.1017/cts.2025.10157

**Published:** 2025-09-26

**Authors:** Jocelyn G. Baker, Kathy Griendling, Lauren A. James, Lillian Eby

**Affiliations:** 1 Department of Psychology, University of Georgia, Athens, GA, USA; 2 Department of Medicine, Emory University School of Medicine, Atlanta, GA, USA; 3 Emory University School of Medicine, Atlanta, GA, USA

**Keywords:** Mentoring, career development, formal mentoring programs, clinical and translational scientists, mentor-mentee matching

## Abstract

**Introduction:**

Mentoring is an important developmental tool for academic scientists. The match between mentor and mentee is a critical factor than can influence outcomes for mentees. However, we know little about what factors should be considered when matching mentors and mentees. We draw on person-environment fit theory to examine how different factors related to the fit between mentors and mentees may influence outcomes for mentees in health-related scientific disciplines.

**Methods:**

Data were collected from 76 mentor-mentee pairs who participated in a nine-month mentoring program for scientists interested in clinical and translational research. An index of supplementary fit was calculated to reflect mentor-mentee similarity in terms of race/ethnicity, gender, academic discipline, professional track, percent time allocated to research, and type of research. Complementary fit reflected the proportion of skills mentees identified as needs that their mentor felt comfortable providing. Mentee outcomes assessed included satisfaction with one’s mentor, learning and development experiences, and short-term research output.

**Results:**

As predicted, we found that supplementary fit was positively related to mentee satisfaction with the mentor. We found no support for the expected relationship between complementary fit and mentee learning development experiences or short-term research output. Supplementary analyses explored other non-hypothesized relationships among study variables.

**Conclusions:**

This research underscores the need to consider different types of fit when matching academic mentors and mentees in clinical and translational science-related disciplines. Our results can be leveraged during the matching process in academic mentoring programs to maximize the success of mentoring relationships for scientists in health-related fields.

## Introduction

Mentoring has the potential to be an invaluable developmental experience for clinical and translational scientists [1–4]. Owing to the importance of mentoring in higher education and Science, Technology, Engineering, Mathematics, and Medicine (STEMM) fields, the National Academies of Sciences, Engineering, and Medicine (NASEM) commissioned a consensus report on the topic to address the gap that exists between what is known about effective mentoring and how it is practiced in higher education and university settings. NASEM describes mentorship as a “professional, working alliance in which individuals work together over time to support the personal and professional growth, development, and success of the relational partners” [5].

The receipt of mentoring has been associated with a host of positive benefits throughout a mentee’s career. For example, mentoring support received by mentees is related to increased satisfaction with and commitment to one’s profession, enhanced learning, lower stress, and lower likelihood of leaving one’s employer [6]. For faculty in STEMM fields in particular, mentoring is associated with higher research activity [7], greater research skill development [8], and faster career progression [9]. Indeed, the NCATS Clinical and Translational Science Awards Program acknowledges that “advancing mentorship […] is key to fostering the growth of a diverse clinical and translational research workforce” [10].

One major decision that is commonly believed to impact mentoring program effectiveness is how to match mentors and mentees [11]. In this research, we explore how different types of *relational fit* between mentees and mentors may contribute to outcomes of interest for mentees working as research scientists exploring topics of relevance to human health. These individuals share some common needs (e.g., grant writing, building a research team, statistical skills) but also need mentoring on discipline-based norms (e.g., manuscript writing, career progression at a specific institution). We draw from the theory and research on person-environment fit, defined as “the compatibility between an individual and a work environment that occurs when their characteristics are well matched” [12]. Although this theory has not been applied to mentoring relationships, meta-analytical research [12] finds that other aspects of person-environment fit (e.g., person–job, person–organization, person–group, and person–supervisor fit) predict a wide range of positive workplace outcomes, such as job attitudes, work performance, retention, psychological well-being and tenure.

The literature on person-environment fit primarily follows two streams: supplementary fit and complementary fit [13]. Supplementary fit refers to fit in terms of similarity. For example, mentor-mentee similarity in terms of race or gender have been shown to be important in the broader mentoring literature [14–15]. When considering mentoring in academic institutions, other types of mentor-mentee similarity may also be important. For example, similarity in academic discipline or amount of time allocated to research may allow the mentor and mentee to approach the relationship with a similar frame of reference. Research on supplementary fit is grounded in Byrne’s similarity-attraction paradigm, which suggests that people are attracted to and develop higher-quality relationships with others with whom they are similar [16–17]. Based on the well-developed body of scholarship on similarity, supplementary fit is likely to be associated with affectively oriented mentee outcomes such as relationship satisfaction [18].

Complementary fit refers to a match in terms of preferences and needs [19]. In the context of mentoring programs in academia, complementary fit would most likely focus on specific skills that mentees may want to develop (e.g., grant writing, building a research team, presentation skills) in relation to what mentors are best suited to provide. Because complementary fit focuses on filling a skill deficit or need, it follows that this type of fit should play an important role in mentee learning and development, as well as research productivity.

Prior research has demonstrated positive mentee outcomes stemming from both supplementary fit and complementary fit. For example, Klaic and colleagues [20] found that supplementary fit with one’s supervisor (e.g., holding similar values) predicts higher job satisfaction and lower work stress. In terms of complementary fit, Ehrhardt and Ragins [21] found that employees’ experience of having their needs met through their relationships at work was associated with a host of positive outcomes such as greater loyalty to their organization, work engagement, and helping behaviors at work, as well as lower turnover and absenteeism. Based on person–environment theory and extending prior empirical research, we propose the following hypotheses:

***Hypothesis 1:*** Supplementary fit (similarity with mentor) is positively related to mentee satisfaction with the mentor.

***Hypothesis 2a:*** Complementary fit (needs-supplies with mentor) is positively related to mentee learning and development experiences.

***Hypothesis 2b:*** Complementary fit (needs-supplies with mentor) is positively related to mentee short-term research output.

It has also been suggested that both types of fit (supplementary and complementary) may interact in predicting positive outcomes [22]. For example, stronger supplementary fit may strengthen the positive effects of complementary fit on mentee outcomes. However, interactive effects of supplementary and complementary fit have not been examined in the context of mentor-mentee relationships or in terms of understanding how to make a good mentor-mentee “match.” Because there is limited prior research upon which to base solid predictions, we pose two exploratory research questions to investigate (1) the possible interactive effects of complementary and supplementary fit on mentee outcomes and (2) the specific aspects of fit that are most strongly associated with mentee outcomes.

***Exploratory Question 1***: Do complementary and supplementary fit interact in predicting mentee outcomes, such that the effects on mentee outcomes (satisfaction with the mentor, learning and development, short-term research output) are strongest when both complementary and supplementary fit are higher?

***Exploratory Question 2:*** Which type of fit is most strongly associated with mentee outcomes?

## Materials and methods

### Program description

Participants consisted of mentor-mentee pairs who participated in the TEAMS (Translational Education and Mentoring in Science) Program offered through the Georgia Clinical and Translational Science Alliance (Georgia CTSA), funded by NCATS [23]. The overall goals of the TEAMS Program were to help mentees (1) develop technical skills for working in a multidisciplinary team science environment, (2) develop relational/interpersonal skills for working in a multidisciplinary team science environment, and (3) develop networks to improve collaboration in multidisciplinary team science. The nine-month program (September–May) provided mentees with the opportunity to meet and interact with colleagues across the four Georgia CTSA institutions (Emory University, Morehouse School of Medicine, University of Georgia, Georgia Institute of Technology). The institutions associated with the Georgia CTSA vary in size (e.g., Morehouse School of Medicine has a current enrollment of around 935 students whereas, Emory has about 7,300 undergraduates, and UGA serves over 40,000 students), funding (e.g., Emory and Morehouse School of Medicine are private universities, whereas UGA and Georgia Tech are public universities), and other characteristics (e.g., UGA is a land grant university, Georgia Tech has an emphasis on STEM education, Morehouse School of Medicine is a historically Black medical school). Participants in the TEAMS program and their mentors represented individuals whose research focuses on human health and who are interested in clinical and translational research. Each participant was matched with a one-on-one mentor who met with them for at least one hour per month (virtually or in-person). In addition to one-on-one mentoring (the focus of this research), mentees were also placed in “learning communities,” with 4–7 other program participants led by a different faculty mentor, which also met monthly to discuss topics of the mentees’ choosing. In addition to one-on-one mentoring and learning community mentoring, mentees participated in a kick-off orientation that included learning about interpersonal interactions, a mid-year professional development session, and program graduation event. A separate study described the development and implementation of the TEAMS program [23]. The current paper includes mentor-mentee matched data from a larger dataset evaluating the TEAMS program, but none of the outcomes examined in this paper have been published elsewhere.

### Recruitment and matching

Faculty, research scientists, clinical fellows, and post-doctoral scholars working on health-related research topics from the four Georgia CTSA institutions were eligible to participate in the program. Faculty from these same CTSA institutions with a history of grant support and working in fields relevant to clinical and translational science were recruited as mentors. Matches were made using application data provided by mentees and mentors. Not only were type of research, research focus, and institution considered, but matching was also performed considering needed relational and technical skill development as assessed by a detailed intake form. For more information see Griendling et al [23].

### Data collection

IRB approval was obtained for the study as secondary data analysis [Protocol #PROJECT00008576]. Both mentors and mentees completed surveys at baseline (pre-program) and at program completion. The current study uses baseline survey data collected online from both mentees and mentors and end-of-program survey data from mentees. The final sample consisted of 76 mentor-mentee pairs with matched data from baseline and end of year, compiled across five different cohorts of the Georgia CTSA TEAMS Program.

The average number of mentor-mentee pairs per cohort was 15.2, ranging from 11–19 pairs. Mentees were primarily female (67.1%) and mentors were primarily male (64.5%). Mentees were 44.7% White, 29.0% Asian, 17.1% Black, 7.9% Latinx, and 1.3% Middle Eastern. Mentors were 65.8% White, 25% Asian, 5.3% Black, 2.6% Middle Eastern, and 1.3% Latinx. Mentors and mentees came from a wide variety of academic disciplines. Among mentees, the most frequently represented academic disciplines were Biomedical Engineering (11.8%), Nursing (9.2%), Oncology (7.9%), and Health Policy & Management (7.9%). For mentors, the most frequently represented academic disciplines were Biomedical Engineering (10.5%), Health Policy & Management (9.2%), Cardiology (9.2%), and Pharmacy (7.9%).

### Supplementary fit

An index of supplementary fit was calculated using data from both mentee and mentor baseline survey data. This index consisted of similarity in terms of race/ethnicity (White, Latinx, Black, Asian, Other), gender (male or female), academic discipline (e.g., Oncology, Psychology, Biomedical Engineering, etc.), and professional track (determined via the question, “Was your mentor someone on the same professional track (e.g., clinician, tenure track faculty) as you?” with response options of yes or no), percent time allocated to research (0%–100%), and type of research (basic, preclinical/translational, clinical, or dissemination and implementation). Table 1 provides a breakdown of the percentage of matches that were similar for each aspect of supplementary fit. Five of the six aspects of similarity (gender, race, academic discipline, research type) were scored 0 (different) or 1 (same). Similarity in the percent time allocated to research was scaled from 0%–100% and then rescaled to 0–1, and the index was formed by summing these six variables. As a result, the final index ranged from 0–6, with 6 indicating the greatest similarity and 0 indicating the least similarity.

**Table 1.** Supplementary information on mentee-mentor matches

Aspect of Supplementary Fit	# Similar
Gender	40/76 (52.6%)
Race	21/76 (27.6%)
Research Type	51/75 (67.1%)
Track	55/74 (72.4%)
Academic Discipline	13/76 (17.1%)
Research Time^*^	*M* = 0.71, *SD* = 0.20

* All aspects of fit except research time were dichotomous. Research time was measured as a percentage (0%–100%) and then rescaled to range from 0–1, with 1 indicating complete similarity in terms of the percentage of one’s time dedicated to research.

### Complementary fit

Complementary fit was determined by counting the number of skills mentees identified as needs that their mentor felt comfortable providing and then dividing by the total number of skills mentees identified as needs (thereby scaling the variable to range from 0–1). On the pre-program survey, mentees were asked to indicate if they would like more training or guidance on a list of skills/topics and mentors were asked to indicate on which skills/topics they felt comfortable providing training or guidance to mentees. Seventeen different skills and topics were included in the calculation of complementary fit, determined through discussion of the mentoring program committee who have a wide range of experience related to faculty development and mentoring program design. The mentoring program committee consisted of representatives from the four institutions that make up the Georgia CTSA (Emory University, Georgia Institute of Technology, Morehouse School of Medicine, and University of Georgia). A mentoring scholar (LE) and a senior faculty member who has previously developed mentoring programs for medical professionals (KG) co-led the committee. The 17 specific skills and topics included in the calculation of complementary fit captured (1) grantsmanship, (2) building and managing research teams, and (3) general skills for professional development. The 17 skills are presented in Table 2.

**Table 2.** Skills examined to determine complementary fit

Category	Skill
Grantsmanship	Grant writing
	Identification of granting agencies and program announcements
	Maximizing preliminary and pilot data
	Choosing the right funding for your career stage
Building and managing research teams	Tips on building/working in/managing an interdisciplinary team
	Finding collaborators
	How to run a meeting
	Managing your research team
	Being an effective team member
	Giving and receiving feedback
	Coaching skills
	Training the next generation
	Building accountability
General skills for professional development	Presentation skills
	Obtaining institutional commitment
	Supporting diversity
	Having difficult conversations

On average, mentees checked 10.64 different skills (standard deviation = 4.09), with a range of 4–17. The top four areas selected by mentees were grant writing (*n* = 63), maximizing preliminary and pilot data (*n* = 61), choosing the right funding for your career stage (*n* = 61), and managing your research team (*n* = 59).

### Mentee outcomes

Mentee outcomes were collected on the survey administered at program completion. Satisfaction with the assigned mentor was assessed using the average of a 2-item self-report measure (1 = Strongly Disagree to 5 = Strongly Agree: “My 1:1 mentor and I developed a high quality relationship,” (*M* = 4.45, *SD* = 0.82) and, “I was satisfied with my 1:1 mentor,” (*M* = 4.49, *SD* = 0.80) adapted from the mentoring literature [25]. The correlation between these two items was 0.90, *p* < 0.001, indicating high internal consistency reliability.

Mentee learning and development experiences were assessed by counting the number of options mentees checked in response to the question, “Have you gained new information/techniques that have enhanced your research as a result of participating in the TEAMS mentoring program? (check all that apply).” The nine response options were a new collaborator, access to new equipment, access to new technique, information about or access to student, patient or community groups, awareness of additional literature, awareness of new funding opportunity, awareness of new resource, creation or discovery of new network, and creation of new team.

Mentee short-term research output was determined by mentee’s responses to the question, “Have you gained any new academic outputs as a result of participating in the TEAMS mentoring program? (Check all that apply),” with response options of Grant, Manuscript, Conference Paper, No, but I anticipate it in the future, or No. Responses were coded as 0 = No, 1 = No, but anticipate it in the future, 2 = Yes (checked one of the research outputs [either Grant, Manuscript, or Conference Paper]), 3 = Yes (checked two of the research outputs), and 4 = Yes (checked all three research outputs).

### Covariates

The TEAMS program also included participation in a learning community. Because we wanted to isolate the effects of the one-on-one mentoring experience, we included a 2-item measure of satisfaction with the learning community as potential control variable. This was measured using the average of the following two items: “I was satisfied with my learning community mentor” and “The learning community component adds value to the TEAMS program” (response options: 1 = Strongly Disagree to 5 = Strongly Agree). The correlation between these two items (*r* = 0.79, *p* < 0.001) supported creating a composite measure of learning community satisfaction.

## Results

Descriptive statistics and correlations among study variables are shown in Table 3. The mean level of supplementary fit was 3.11 (*SD* = 1.23) out of a maximum of 6. The mean level of complementary fit was 0.60 (*SD* = 0.29) out of a maximum score of 1. This indicates that on average, mentor-mentee pairs had moderate levels of both supplementary and complementary fit in terms of the variables we explored.

**Table 3.** Descriptive statistics and intercorrelations

	M	SD	1	2	3	4	5	6	7	8	9	10	11
Satisfaction with Mentor (1)	4.47	0.79	0.90										
New Experiences (2)	2.29	1.59	0.17	1									
Short-Term Research Output (3)	1.11	0.87	−0.01	0.36^**^	1								
Fellow Learning Community Satisfaction (4)	4.59	0.69	−0.06	0.19	0.36^**^	0.79							
Gender Similarity (5)	0.53	0.50	0.26^*^	0.04	0.02	0.16	1						
Race Similarity (6)	0.28	0.45	0.13	−0.04	−0.14	−0.10	−0.06	1					
Academic Discipline Similarity (7)	0.17	0.38	0.17	0.18	0.11	0.05	0.15	−0.05	1				
Research Time Similarity (8)	0.71	0.20	0.06	−0.04	0.12	0.13	0.00	0.08	−0.12	1			
Research Type Similarity (9)	0.68	0.47	0.04	0.09	0.23^*^	−0.06	0.05	−0.04	0.31^**^	−0.08	1		
Professional Track Similarity (10)	0.74	0.44	−0.03	−0.01	−0.13	−0.16	0.00	0.16	0.27^*^	−0.04	0.32^**^	1	
Complementary Fit (11)	0.60	0.29	0.06	0.13	0.21	0.05	0.19	0.02	−0.12	−0.08	0.18	−0.02	1
Supplementary Fit (12)	3.11	1.23	0.26^*^	0.09	0.06	−0.01	0.44^**^	0.40^**^	0.52^**^	0.12	0.62^**^	0.61^**^	0.09

*Note*. Dichotomous variables (gender, race, academic discipline, research type, and professional track similarity) were coded such that 1 = match between mentor and mentee, 0 = not a match between mentor and mentee. Coefficients on the diagonal represent inter-item correlations for 2-item measures. *M* = mean, *SD* = standard deviation. ^*^indicates *p* < 0.05. ^**^indicates *p* < 0.01.

In terms of outcomes, the average level of satisfaction with one’s mentor was 4.47 (*SD* = 0.79, range 1–5). The mean level of mentee short-term research outputs was 1.11 (*SD* = 0.87, range 0–3). For mentee learning and development experiences, on average, mentees endorsed 2.29/9 items (*SD* = 1.59, range = 0–6). The most frequently selected items reflecting mentee learning and development experiences were awareness of new funding opportunity (*n* = 36), a new collaborator (*n* = 31), awareness of new resource (*n* = 30), and creation or discovery of new network (*n* = 28).

Learning community satisfaction was positively related to short-term research output (*r* = 0.36, *p* < .01), supporting the decision to statistically control for this variable in hypothesis testing [26]. Supplementary analyses excluding satisfaction with the learning community yielded the same pattern of results and are available upon request. Hypotheses 1–2 were tested using linear regression in SPSS (controlling for mentee satisfaction with their learning community). Hypothesis 1, which predicted that supplementary fit is positively related to mentee satisfaction with the mentor, was supported, *β* = 0.26, *p* = 0.04, *F*(2) = 2.17, R^2^ = 0.07. Hypothesis 2a, which predicted that complementary fit is positively related to mentee learning and development experiences, was not supported, *β* = 0.12, *p* = 0.36, *F*(2) = 1.17, R^2^ = 0.04. Hypothesis 2b, which predicted that complementary fit is positively related to mentee short-term research output, was not significant but trending in the expected direction, *β* = 0.19, *p* = 0.12, *F*(2) = 5.28, R^2^ = 0.15.

Exploratory question 1, which investigated if complementary and supplementary fit interacted in predicting mentee outcomes, was tested using the PROCESS macro for moderation (Model 1) in SPSS. There was no significant interaction between complementary and supplementary fit in predicting any of the outcomes (satisfaction with mentor: *p* = 0.85, *F*(4, 54) = 1.02, R^2^ = 0.07; mentee learning and development experiences; *p* = 0.21, *F*(4, 54) = 1.07, R^2^ = 0.07, mentee short-term research outputs: *p* = 0.27, *F*(4, 54) = 2.53, R^2^ = 0.16). To answer exploratory question 2, which asked which type of fit is most strongly correlated with mentee outcomes, we examined correlations (see Table 3). The specific type of supplementary fit that had the strongest (and the only significant) relationship with satisfaction with the mentor was gender similarity, *r* = 0.26, *p* = 0.03, such that greater gender similarity was related to higher satisfaction with the mentor. Table 3 also shows that short-term research output was positively related to mentee learning and development opportunities (*r* = 0.36, *p* < 0.01).

## Discussion

In summary, our research illustrates that mentor-mentee similarity may be an important factor to consider when matching scientists interested in clinical and translational research in mentoring relationships. This aligns with existing literature emphasizing the importance of similarity in mentoring relationships [6,24,25]. In particular, the combination of several factors such as gender, race, and academic discipline similarity may collectively contribute to increasing the mentees’ satisfaction with their assigned mentor.

Although gender similarity was the aspect of supplementary fit that had the strongest relationship with mentee satisfaction with one’s mentor, we acknowledge other research finding that deeper-level similarity variables (e.g., attitudes, beliefs, values) are more important in predicting mentoring outcomes compared to surface-level similarity variables (e.g., gender, race, ethnicity) [27].

“Surface-level” and “deep-level” are psychological terms from organizational science referring to different types of diversity, where “surface-level” refers to characteristics that tend to be easily observable, such as most demographic variables and “deep-level” refers to characteristics that are not immediately observable but may become apparent over time, such as personal and professional attitudes and values [28]. Other research in mentoring in STEMM fields has found stronger effects for perceived similarity (e.g., perceptions of sharing similar outlooks, perspectives, and values) compared to demographic similarity [29]. That being said, much remains to be learned about how similarities and differences in each type of social identity (surface and deep level) may impact mentoring relationships in the sciences, and future research should continue to explore this important topic [30].

Furthermore, it is possible that matching on gender similarity may have unintended negative consequences for female mentors by placing additional service-type burdens on established female scientists [31]. One alternative to matching based on gender might be to foster closeness in other ways. Two promising approaches include relational interventions that foster psychological similarity [32] or self-expansion (increasing resources, perspective, and identities that facilitate personal goal achievement [33] through mentee-mentor training that provides structed opportunities for progressive self-disclosure [34]. We encourage future research to explore whether similarity in attitudes (e.g., motivation for research, perceived utility of scientific knowledge), beliefs (e.g., methodological philosophy, epistemological beliefs), and values (e.g., objectivity, research integrity, curiosity) of potential relevance to academic mentees and mentors may likewise predict mentee outcomes.

Additionally, having needs-supplies complementary fit may be important in fostering research productivity. Although this positive relationship was not statistically significant, several factors likely limited our ability to find statistically significant support for our hypotheses. First, research output (e.g., grants, manuscripts) often take considerable time (e.g., years) to manifest, and the mentoring program took place over the course of nine months. Future research should track mentee outcomes over longer periods of time. Our small sample size and subsequent lack of statistical power also likely contributed to the lack of significance. Furthermore, although our operationalization of complementary fit considered which skills mentees wanted guidance on and which skills mentors felt comfortable offering to mentees, we do not know if the skills were the focus of individual mentoring relationships. This latter point may also offer insight into why complementary fit was not related to mentee learning and development opportunities in this study.

An important caveat in interpreting our findings is that the operationalization of both research outputs and mentee learning and development experiences asked mentees to report if they gained these outputs as a result of the TEAMS program, not specifically their one-on-one mentoring relationship. As such, we are unable to isolate which specific experiences mentees gained from their mentor as opposed to other parts of the TEAMS program, such as the learning community, skills development workshops, or informal networking that may have occurred during participation in the program. To address this issue, we examined open-ended comments in response to the question, “What did you find most beneficial about your participation in the TEAMS program.” Sixty-nine of the 79 participating fellows (87.3%) provided open-ended comments to the question. Of those that provided comments, 48.1% mentioned one-on-one mentoring, 29.1% mentioned their learning community, and 17.7% mentioned both the one-on-one and learning community experience. We also found that 39.2% specifically mentioned benefits related to learning and development or research output (e.g., “[benefits included] the 1:1 mentorship and formation of team groups with individuals in similar stages of career development having open conversations and ability to learn from each other;” “My mentor was nothing short of exceptional: he was encouraging, always had time to lend an ear about projects or even just to vent, and had practical/meaningful advice to give for nearly every situation I faced this year’). These post-hoc analyses suggest that learning occurred through both the one-on-one mentoring relationship and in the learning community but was more often noted as a benefit of the one-on-one component of the program.

These findings have practical implications for developing the next generation of scientists interested in improving human health and for mentoring program design. When designing mentoring programs, leaders should carefully consider the mentee outcomes to target. Satisfaction with one’s mentor (and relationship quality more broadly) is often considered an important outcome in mentoring programs [6]. Interestingly, we conducted a post-hoc analysis and found that satisfaction with one’s mentor had a near-zero relationship with short-term research output (*r* = −0.01, *p* = 0.96), which may also be an outcome of interest. This suggests that different factors may be important to consider when designing mentoring programs intending to increase both types of outcomes. Furthermore, it may be worth considering supplementing a one-on-one mentoring program with a ‘learning community,’ as satisfaction with one’s learning community was significantly related to short-term research output. This aligns with other research suggesting the benefits of group or peer mentoring [35–37].Future research could explore more deeply the benefits of group mentoring in combination with traditional one-on-one mentoring for scientists in health-related fields.

In terms of generalizability, one unique feature of the TEAMS program is that it focuses on developing professional skills in the areas of translational and clinical research, with special emphasis on multidisciplinary teams and team science (https://georgiactsa.org/research/cmdts/mentoring/index.html). Given this focus, the program does not match mentors and mentees on “perfect research fit.” Rather, we identified points of potential research intersection and based matching more on the opportunity for participants to broaden their understanding of building and managing multidisciplinary teams and developing competencies related to effectiveness in this role. Because of the TEAMS program’s emphasis, participants self-selecting into the program may differ from mentees in other university-sponsored mentoring programs due to their interest in team science and professional development orientation. In conclusion, the present study highlights the importance of considering different types of fit when matching faculty mentors and mentees working in research areas related to human health. While similarity is one important factor, having complementarity in terms of the mentor being able to meet the mentees’ needs is also worth consideration. Although there is no perfect formula to guarantee a successful mentoring relationship, these findings can be applied to increase the likelihood of a successful match and to contribute to the career development of faculty in health-related academic disciplines.
